# Topical Treatment of Recurrent Vulvovaginal Candidiasis: An Expert Consensus

**DOI:** 10.1089/whr.2021.0065

**Published:** 2022-01-31

**Authors:** Nancy A. Phillips, Gloria Bachmann, Hope Haefner, Mark Martens, Colleen Stockdale

**Affiliations:** ^1^ Department Obstetrics, Gynecology and Reproductive Sciences, Rutgers Robert Wood Johnson Medical School, New Brunswick, New Jersey, USA.; ^2^ Department Obstetrics and Gynecology, University of Michigan University Hospital, Ann Arbor, Michigan, USA.; ^3^ Department Obstetrics and Gynecology, Tower Health, West Reading, Pennsylvania, USA.; ^4^ Department Obstetrics and Gynecology, University of Iowa, Iowa City, Iowa, USA.

**Keywords:** consensus review, recurrent yeast, vulvovaginitis, candidiasis, topical therapy

## Abstract

***Background:*** Recurrent vulvovaginal candidiasis (RVVC), defined as three or more confirmed infections over 1 year, occurs in up to 10% of women. In these women, the objective is often symptomatic control rather than mycologic cure. Current Centers for Disease Control and Prevention (CDC) guidelines recommend oral fluconazole as first-line maintenance, but state if this oral regimen is not feasible, intermittent topical treatments can be considered. No specific recommendations for type or frequency of topical applications are provided by the CDC.

***Methods:*** A panel of vulvovaginal experts convened to develop a consensus recommendation for topical maintenance dosing for RVVC.

***Results:*** Data suggest that clotrimazole, miconazole, terconazole, and intravaginal boric acid are suggested recommendations for recurrent vulvovaginitis caused by both *Candida albicans* and nonalbicans species. Nystatin ovules may not be as effective as azoles. Identification of species will influence treatment decisions. In addition, treatment may be modified based on prior response to a specific agent, especially in nonalbicans species. Fluconazole, ibrexafungerp, and intravaginal boric acid should be avoided during pregnancy.

***Conclusions:*** The expert consensus for women with RVVC is an initial full course of treatment followed by topical maintenance beginning at one to three times weekly, based on chosen agent. Twice a week dosing was the regimen most often utilized. In some women, episodic treatment may be used, but maintenance should remain an option for this population.

## Introduction

Vulvovaginal candidiasis (VVC) occurs when a Candida species causes a local inflammatory response, resulting in itching, irritation, burning, and/or dyspareunia. Up to 49% of women over the age of 16 years and 55% of college-aged women report having had at least one previously clinically diagnosed episode of VVC.^1^ Data suggest that recurrent vulvovaginal candidiasis (RVVC) (previously defined as four or more infections in <1 year) occurs in 6%–10% of women.^2^ This percentage may increase, as the definition has been changed recently to three or more infections over 1 year.^3^

The Centers for Disease Control and Prevention (CDC) guidelines for treatment of uncomplicated VVC (Table 1) include short-course topical formulations, available over the counter (OTC) or by prescription or a single dose of oral fluconazole. These therapies result in relief of symptoms and negative cultures in 80%–90% of women who complete therapy.^3^

**Table 1.** **2015 Centers for Disease Control and Prevention Recommendations**^a^ **for Acute Treatment of Uncomplicated**^b^ **Vulvovaginal Candidiasis**

Over-the-counter intravaginal agents	
Clotrimazole 1% cream	5 g intravaginally daily for 7–14 days
Clotrimazole 2% cream	5 g intravaginally daily for 3 days
Miconazole 2% cream	5 g intravaginally daily for 7 days
Miconazole 4% cream	5 g intravaginally daily for 3 days
Miconazole 100 mg vaginal suppository	One suppository daily for 7 days
Miconazole 200 mg vaginal suppository	One suppository for 3 days
Miconazole 1,200 mg vaginal suppository	One suppository for 1 day
Tioconazole 6.5% ointment 5 g intravaginally	In a single application
Prescription intravaginal agents	
Butoconazole 2% cream bioadhesive product	5 g single intravaginal application
Terconazole 0.4% cream	5 g intravaginally daily for 7 days
Terconazole 0.8% cream	5 g intravaginally daily for 3 days
Terconazole 80 mg vaginal suppository	One suppository daily for 3 days
Oral agent	
Fluconazole^c^ 150 mg orally in a single dose	

Adapted from CDC Sexually Transmitted Infections Treatment Guidelines, 2021 (https://www.cdc.gov/std/treatment-guidelines/candidiasis).

^a^ New CDC guidelines being released in late 2021.

^b^ Uncomplicated: sporadic or infrequent VVC and mild-to-moderate VVC and likely to be *Candida albicans* and nonimmunocompromised women.

^c^ Fluconazole should be avoided during pregnancy.

CDC, Centers for Disease Control and Prevention; VVC, vulvovaginal candidiasis.

In women with RVVC, mycologic cure is more difficult to achieve. To maintain clinical and mycologic control, a longer duration of initial therapy (*e.g.*, 7–14 days of topical therapy or a 100-, 150-, or 200-mg oral dose of fluconazole every third day for a total of three doses [days 1, 4, and 7]) is recommended, to attempt mycologic remission, before initiating a maintenance antifungal regimen. (Maintenance therapy for control of clinical symptoms rather than treatment of each episode of VVC is recommended.) Confirmation of RVVC and identification of species are important to guide therapy.

The CDC guidelines recommend oral fluconazole (*i.e.*, 100-, 150-, or 200-mg dose) weekly for 6 months as the first-line maintenance regimen in RVVC. The CDC also states that if this oral regimen is not feasible, topical treatments used intermittently can also be considered.^3^ However, the CDC does not provide recommendations for the type or frequency of topical maintenance, leaving clinicians without templates regarding best practice.

Nor does the CDC define when oral fluconazole is not feasible. Women generally prefer an oral over topical regimen based on convenience, and fluconazole should remain the treatment of choice in appropriate candidates. However, the need for topical maintenance therapies is based on several limitations of oral fluconazole suppression. Fluconazole use should be avoided or closely monitored in women on statin drugs, and those with kidney disease or at high risk for arrhythmias. Other potential drug interactions should also be checked before prescribing fluconazole.^4^

In addition, according to a 2015 nationwide cohort study from Denmark, women who had taken fluconazole during pregnancy were at a significantly increased risk of miscarriage compared with those with no exposure to fluconazole and with women who had used a topical azole drug.^5^ This should be considered with the use of fluconazole in women of childbearing age. Importantly, fluconazole may not be effective against nonalbicans species, which are estimated to be responsible for up to 20% of RVVC. *Candida glabrata* is thought to be responsible for most nonalbicans species, with less virulent species such as *Candida parapsilosis* and *Candida krusei* responsible for a smaller percentage.^6^

In June 2021, ibrexafungerp was approved by the Food and Drug Administration (FDA). Interestingly, this medication is fungicidal, compared with fluconazole that is fungistatic. It is dosed at 300 mg by mouth twice in 1 day. It does not cause liver toxicity and its activity is not impacted by a low vaginal pH. It is active against *C. glabrata*. Ibrexafungerp is contraindicated during pregnancy.

The International Society for the Study of Vulvovaginal Disease (ISSVD) has an app (The Vulvovaginal Candidiasis [Candida, Yeast]: Tips for Diagnosis and Treatment app, available on iPhone) that provides guidance for treatment of RCVV by species. This underscores the importance of not only confirming yeast as the cause of the disorder, but also identifying the species before initiating long-term therapy.

Additional considerations for management of women with RCVV include exploring and altering or eliminating factors that may promote the infection. This may include removing intrauterine devices in some women, improved control of diabetes, and optimizing immunocompromised states. Review of medications, with elimination of sodium glucose cotransporter two inhibitors (which promote glycosuria) if possible, and minimizing, when feasible, use of corticosteroids and antibiotics may be helpful in long-term control of RCVV.

## Materials and Methods

A panel of vulvovaginal experts was convened by the sponsors (Prestige Consumer Healthcare), based on their clinical and research experience, to develop a consensus article on recommendation for topical dosing in RVVC maintenance. Before the meeting, participants were encouraged to review the CDC guidelines and the ISSVD consensus guidelines. They were also expected to be up to date on the peer-reviewed literature and perform a literature review for the treatment of RVCC.

## Results

Review of available data suggests that clotrimazole, miconazole, terconazole, and intravaginal boric acid are the suggested interventions for RVVC caused by *Candida albicans*. Nystatin ovules remain an option for treatment in most cases of RVVC, but the CDC states that azoles may be more effective^3^^,^^7––9^ (Table 2). In addition, nystatin ovules are currently not available in the United States and need to be compounded by specialty pharmacies. For *nonalbicans* RVVC, the same interventions were recommended, although some experts felt a nonterconazole azole should be used unless prior response to terconazole has been demonstrated (Table 3).

**Table 2.** Recommendations: Topical Drugs and Dosing Regimens for Maintenance Recurrent Vulvovaginal Candidiasis (*Candida albicans*)

Management options
Three-day treatments (azoles): clotrimazole 2% cream; miconazole 4% cream/200 mg suppositories/200 mg ovule; terconazole 0.8% cream
Seven-day treatments (azoles): clotrimazole 1% cream; miconazole 2% cream; terconazole 0.4% cream
Boric acid: 600 mg suppositories
Nystatin: 100,000-unit ointment; 100,000-unit ovules
Use the listed management options (6 months) 1–3 times weekly to start, then maintain or reduce frequency of dosing depending on the frequency of recurrence.
Most often recommendation is twice a week dosing.
If using a 3-day strength formulation, consider once a week dosing
If using a 7-day strength formulation, consider twice a week dosing
Notes
Before maintenance therapy, a full course of therapy is recommended
Only 7-day azoles are recommended during pregnancy
Patients with significant symptoms who are being treated with oral fluconazole may benefit from topical azoles (alone or in combination with a steroid)
Boric acid precautions should always be reviewed

**Table 3.** Recommendations Topical Drugs and Dosing Regimens Maintenance Recurrent Vulvovaginal Candidiasis (*Nonalbicans Species*)

Management options
Three-day treatments (azoles): clotrimazole 2% cream; miconazole 4% cream/200 mg suppositories/200 mg ovule; terconazole 0.8% cream^a^
Seven-day treatments (azoles): clotrimazole 1% cream; miconazole 2% cream; terconazole 0.4% cream^a^
Boric acid 600 mg suppositories
Use the listed management options (6 months) 1–3 times weekly to start, then maintain or reduce frequency of dosing depending on the frequency of recurrence.
Most often recommendation is twice a week dosing.
If using a 3-day strength formulation, consider once a week dosing
If using a 7-day strength formulation, consider twice a week dosing
Notes
Before maintenance therapy, a full treatment course is recommended
Only 7-day azoles are recommended during pregnancy
Boric acid precautions should always be reviewed

^a^ Some experts recommend a nonterconazole agent should be considered unless prior susceptibility to terconazole has been demonstrated.

According to this expert consensus, after the initial treatment has been completed, for recurrent infections, topical dosage and frequency should begin at one to three times weekly, then maintained or reduced in frequency of dosing depending on the frequency of recurrence. Twice a week dosing was the regimen most often utilized. Intravaginal boric acid is as an option for either *C. albicans* or *nonalbicans* species, but is not as frequently utilized in *C. albicans* where azole coverage is generally successful. During pregnancy, a 7-day azole is recommended for initial dosing, and boric acid, fluconazole, and ibrexafungerp should be avoided (See Supplementary Box S2). Recommendations for the effective topical agents for individual species can be found in the ISSVD VVC—yeast app (app store, ISSVD).

## Discussion

Literature concerning suppressive or maintenance therapy with topical treatments is limited. Research by Fong explored the treatment of 23 women with a 500 mg dose of clotrimazole monthly with menses for 6 months versus one dose with onset of symptoms, with a crossover after 6 months. Fong showed that women treated prophylactically developed 2.2 episodes of VVC versus 3.6 in the empiric group. The women in this trial preferred symptomatic treatment only.^7^ Stein et al. used terconazole 0.8% cream initially for a symptomatic episode of candida vaginitis, followed by weekly applications of 0.8% cream for 26 weeks in 22 women with RVVC.

Their data showed there were statistically significant less infections during the prophylactic period than the 26-week follow-up period (4 vs. 14).^8^ A 2000 review in American Family Physician recommended clotrimazole two 100-mg tablets administered intravaginally twice weekly for 6 months or terconazole 0.8% cream one full applicator (5 g) administered vaginally once a week.^9^ Since that time, *in vitro* studies suggest less effectiveness of terconazole against *nonalbicans* species, especially at physiologic pH levels.^10^

Boric acid has limitations that need consideration (Supplementary Box S1). Boric acid is not FDA approved. Until recently with the introduction of OTC boric acid products, it needed to be compounded. Also, there are no long-term safety data available for vaginal use. Boric acid can be toxic if ingested orally. The National Pesticide Information Center characterizes the toxicity risk as low with the most common side effects being nausea, vomiting, and diarrhea, with fatality being rare.^11,12^ Nonetheless, clinicians should advise care with storage of boric acid capsules, away from orally ingested medications and out of reach of children and pets. In addition, during use, oral sex should be avoided. Boric acid is contraindicated during pregnancy.

Recent studies have shown that at different pH values, antifungals may have different minimum inhibitory concentrations (MICs) with a lower MIC indicating better effectiveness and less resistance. *In vitro* testing is often at a pH of 7, whereas the normal vaginal pH is 4. When *in vitro* testing is performed at this lower more physiologic pH, MIC values of antifungals have been shown to be higher.^13^

A 2018 retrospective analysis of 217 first positive yeast cultures from 217 patients confirmed a higher MIC for all antifungals with *in vitro* testing at pH 4.^10^ For *C. albicans* the largest MIC difference was for terconazole (0.17 pH 7 vs. 6.17 pH 4) and clotrimazole (0.04 vs. 0.24). When testing antifungal MIC against *C. glabrata* at the lower pH (4 versus 7) terconazole was most affected (0.26 at pH 7 vs <64 at pH 4). Clotrimazole (0.13 vs 6.96), miconazole (0.06 vs 0.76) and fluconazole (3.17 vs 26.6) also showed a higher MIC against *C. glabrata* at the lower pH. Miconazole was least affected with MIC remaining <1.^10^

Although clinical correlation with these *in vitro* studies is limited, these data reinforce that before suppressive therapy is initiated, the agent used should be evaluated for efficacy.

The duration of suppressive therapy is generally recommended for 6 months. However, 30%–50% of women will recur after maintenance therapy is discontinued. Women who recur after initial suppression or for those who culture positive during suppressive therapy should be referred to a health care provider with experience in managing RVVC.

## Conclusion

Expert consensus on for the topical treatment of RVVC supported maintenance therapy with clotrimazole, miconazole, terconazole, or boric acid caused by *C. albicans*. For *nonalbicans* RVVC, the same interventions were recommended, although some experts felt a nonterconazole azole should be used unless prior response to terconazole had been demonstrated. After the initial treatment has been completed, maintenance should begin at one to three times weekly, based on chosen agent, then maintained or reduced in frequency of dosing depending on the frequency of recurrence. Twice a week dosing was the regimen most often utilized.

These recommendations can be used to guide practicing clinicians. Although for most women fluconazole suppression will be adequate, clinical decisions should be based on identification of yeast species, patient characteristics, and concomitant medication and pregnancy plans. In some women, episodic treatment is an option, but maintenance should also be considered for this population.

## Disclaimers

The views expressed in this article are those of the authors and not an official position of any of the associated institutions or the funder.

## Supplementary Material

Supplemental data

Supplemental data

## Supplementary Material

Supplementary Box S1

Supplementary Box S2

## Abbreviations Used

CDC: Centers for Disease Control and Prevention
FDA: Food and Drug Administration
ISSVD: International Society for the Study of Vulvovaginal Disease
MIC: minimum inhibitory concentrations
OTC: over the counter
RVVC: recurrent vulvovaginal candidiasis
VVC: vulvovaginal candidiasis
