# Supplementary material for: Topical Treatment of Recurrent Vulvovaginal Candidiasis: An Expert Consensus
Source: Womens Health Rep (New Rochelle). 2022 Jan 31;3(1):38–42. doi: 10.1089/whr.2021.0065 (PMC8812501; doi:10.1089/whr.2021.0065)
Supplement: Supplemental data [file Suppl_BoxS1.docx]

Box 1: Boric Acid Precautions

Boric acid is poisonous it taken orally

Treatment should always be intravaginal

Keep Boric Acid tablets secured away from other oral medications

Keep Boric Acid tablets secured away from children and pets

Avoid all sex, but most importantly oral sex, during treatment
